# Mitochondria and Coenzyme Q10 in the Pathogenesis of Preeclampsia

**DOI:** 10.3389/fphys.2018.01561

**Published:** 2018-11-15

**Authors:** Enrique Teran, Isabel Hernández, Leandro Tana, Santiago Teran, Carlos Galaviz-Hernandez, Martha Sosa-Macías, Gustavo Molina, Andrés Calle

**Affiliations:** ^1^Colegio de Ciencias de la Salud, Universidad San Francisco de Quito, Quito, Ecuador; ^2^Facultad de Enfermería, Pontificia Universidad Católica del Ecuador, Quito, Ecuador; ^3^Instituto Politécnico Nacional, Durango, Mexico; ^4^Facultad de Ciencias Médicas, Universidad Central del Ecuador, Quito, Ecuador

**Keywords:** preeclampsia, placenta, mitochondria, coenzyme Q10, pregnancy

## Abstract

Hypertensive disorders during pregnancy constitute one of the main causes of maternal and perinatal morbidity and mortality across the world and particularly in developing countries such as Ecuador. However, despite its impact on public health, the primary pathophysiological processes involved are yet to be elucidated. It has been proposed, among other theories, that an abnormal placentation may induce an endothelial dysfunction, which is ultimately responsible for the final clinical manifestations. Mitochondria, particularly from trophoblastic cells, are responsible for the production of energy, which is extremely important for normal placentation. The malfunction in this supply of energy may produce higher levels of free radicals. In both production of energy and free radicals, coenzyme Q10 (CoQ10) plays a crucial role in electron transport. As such, the role of CoQ10 in the genesis and prevention of preeclampsia has become the focus of a number of research groups, including that of the authors. Developing an in-depth understanding of these mechanisms might allow us to design new and feasible strategies with which we can reduce preeclampsia, particularly in the Latin-American countries.

## Introduction

Maternal mortality remains a significant public health problem in Ecuador (The Maternal Health Study [MNPI], 1999; Centro de Estudios de Población y Desarrollo Social [CEPAR], 2004; Instituto Nacional Ecuatoriano de Estadísticas y Censos [INEC], 2017). The main cause for maternal mortality is preeclampsia – a disease characterized by hypertension, increased vascular resistance, endothelial dysfunction, proteinuria, and coagulopathy – that usually manifests in the second trimester of pregnancy (National High Blood Pressure Education Program Working Group Report on High Blood Pressure in Pregnancy, 1990). Preeclampsia is defined as an increase in blood pressure that is equal to or greater than 140/90 mmHg and 24 h of proteinuria equal to or greater than 300 mg/dl (Solomon and Seely, 2001). The presence of preeclampsia during pregnancy also causes complications to the newborn and is associated with low birth weight, preterm delivery, and neonatal mortality (López-Jaramillo, 1993). However, despite several years of basic research, the genesis and the pathophysiology of preeclampsia still remain unknown (Armaly et al., 2018).

For several years, we have dedicated our research efforts toward acquiring a better understanding of preeclampsia and designing new strategies, concepts, and hypotheses that might allow us to unlock the enigma of the etiology of preeclampsia (Calle and Teran, 2018). In this paper, we describe the evidence that highlights the putative role of abnormalities in placental implantation and, more specifically, in terms of mitochondrial function at the placental level on the genesis of preeclampsia. The effects of these alterations upon the maternal endothelium are discussed elsewhere (Roberts and Redman, 1993; Evora, 2000; Granger et al., 2001; Saito and Nakashima, 2014).

## Development of the Placenta During Normal Pregnancy and in Preeclampsia

The placenta is a temporary organ that forms a physical and functional connection between the mother and the embryo/fetus during development. However, in contrast to other organs, the environment in which the placenta develops during the first 8–10 weeks is poor in oxygen; it has been demonstrated that this is essential for blastocyst implantation and further embryo and placental development (Genbacev et al., 1997; Goldman-Wohl and Yagel, 2002).

The trophoblast cells differentiate into the embryo and are derived from the trophectoderm and develop into the blastocyst during development; these cells are essential in maintaining the further development of a normal pregnancy (Genbacev et al., 1997). The trophoblast adheres to the uterus and begins to penetrate into the endometrial stroma. Previous research has shown that proteolytic enzymes such as metalloproteinase act as facilitators in the penetration process by degrading the extracellular matrix (Goldman-Wohl and Yagel, 2002).

The implantation of the blastocyst and primitive placental formation are associated with active penetration of the trophoblast into the endometrial epithelium and adjacent stroma in a low-pressure oxygen environment, which facilitates this process (Genbacev et al., 1997). Subsequently, when the trophoblast is in an environment of high oxygen pressure (Genbacev et al., 1997), it begins the process of differentiation from an early proliferative to an invasive phenotype that will continue to penetrate up to the first third of the myometrium (Zhou et al., 1997b). Interestingly, these cells can also invade the maternal spiraled arteries, which helps to refurbish and replace endothelial and muscular cells (Zhou et al., 1997b). This procedure, known as the conversion, is the replacement of the fetal by maternal endothelium and a loss of elasticity in the arterial vessels. Arteries, then by losing their elasticity, become more compliant, like veins, to increase its capability, according to the demand during pregnancy. In this way, blood flow increases in order to supply nutrients and oxygen to the embryo/fetus during development.

The syncytiotrophoblast is responsible for nutrient exchange, and it is derived from the fusion of the trophoblast cells, which creates an impermeable cellular wallpaper (Goldman-Wohl and Yagel, 2002). In addition to the maintenance of the placental nutrients, the syncytiotrophoblast is also responsible for gaseous exchange as well as the production of hormones and growth factors.

Dependence on oxygen is necessary for normal placental development (De Marco and Caniggia, 2002). The processes underlying the differentiation and the invasion of the trophoblast into the endometrium consume significant amounts of energy (Widschwendter et al., 1998). Based on this requirement, placental development requires an organ acting as an oxygen sensor to modulate energy production. Experimental studies have demonstrated that mitochondria and, more specifically, respiratory chain complexes appear to be responsible for such regulation (De Marco and Caniggia, 2002).

To determine the mechanisms responsible for how and why the placenta and trophoblastic cells can detect oxygen levels may have significant clinical relevance because preeclampsia or the retardation of intrauterine growth involves changes in placentation.

## Abnormal Placentation During Preeclampsia

Several studies have shown that an alteration in placental function is linked to the development of preeclampsia. This pathology can occur in pregnancies without a fetus being present, for example, in the condition referred to as mole hydatidiform (Page, 1939) or via poor placentation (Goldman-Wohl and Yagel, 2002) and thus cause an increase in the placental mass, which is the characteristic of gestational diabetes (Solomon and Seely, 2001) or multiple pregnancies (Thornton and Macdonald, 1991). Notably, preeclampsia resolves after delivery (Redman, 1991). Furthermore, histopathological studies have demonstrated morphological changes in vessels from preeclamptic placentas in a process that has come to be known as “endotheliosis” (Gerretsen et al., 1981). A number of experimental studies have analyzed these observations (Gerretsen et al., 1981; Redman, 1991; Colburn et al., 1994; Graham and McCrae, 1996; Lim et al., 1997; Zhou et al., 1997a) and have noted that during preeclampsia, there are two functional abnormalities: first, invasion of the trophoblast to the uterine parenchyma is not deeper and invasion to the vasculature does not reach up to the decidua portion and into the spiral arteries. As a consequence, maternal vessels do not complete the normal physiological changes known as conversion. Consequently, the diameter of the myometrial vessels in preeclampsia is less than half of those in normal pregnancy (Brosens et al., 1972); furthermore, these vessels maintain their responsiveness to vasoconstrictors such as angiotensin or epinephrine (van der Graaf et al., 2013). Second, the number of vessels showing evidence of trophoblastic invasion is reduced in comparison with normal pregnancy (Khong et al., 1986).

However, there has been significant debate over the potential causes of such alterations. An immunological hypothesis targets a potential maternal sensitization process against fetal tissue. It has been shown that longer sexual cohabitation (Dekker and Baha, 2001) and sexual practices that expose the maternal environment to the partner’s semen, for example, oral sex or intercourse without a condom (Koelman et al., 2000), can reduce a women’s risk for the further development of preeclampsia (Saftlas et al., 2014).

In the maternal decidua, there is a significant concentration of leucocytes; of these, the most important are the natural killers (NK cells). During preeclampsia, the trophoblast experiences an increased level of cellular lysis by maternal NK cells (Colburn et al., 1994), which is responsible for an abnormal sensitization process. Therefore, during preeclampsia, there is a reduction in the expression of human leucocyte antigen G (HLA-G) in the invasive trophoblast (Colburn et al., 1994), making these cells more susceptible to attack by NK cells present in the maternal decidua. This process seems to be maintained throughout pregnancy, meaning that trophoblastic cells do not have the resources to support appropriate implantation. In these cases, the production of lysis enzymes in the extracellular matrix from the maternal decidua is abnormal (Lim et al., 1997) and the enzymes produced have structural problems and, therefore, are not able to function appropriately (Graham and McCrae, 1996). Consequently, the trophoblast cannot break down the maternal extracellular matrix and, hence, invasion is not possible.

In addition, during the process of placentation, several factors, such as growth factors and intercellular adhesion molecules, control trophoblast differentiation and also the process of conversion. During preeclampsia, these factors and their functions are often abnormal; thus, trophoblast invasion and conversion are also abnormal (Zhou et al., 1997a; Goldman-Wohl and Yagel, 2002).

Finally, for all these cellular processes, it is known that the trophoblast requires oxygen to produce energy at the mitochondrial level (Genbacev et al., 1997; De Marco and Caniggia, 2002). Several reports have been now published showing an abnormal mitochondrial function in the trophoblast cells during preeclampsia (Furui et al., 1994; Vuorinen et al., 1998; Wang and Walsh, 1998; Roberts and Lían, 2002).

## Mitochondria and Preeclampsia

Mitochondria are intracellular organelles responsible for energy production through the respiratory chain in a process that uses oxygen to form adenosine triphosphate (ATP), which is known as oxidative phosphorylation (Davidson, 1994). This process is characterized by the transport of electrons from NADH and FADH_2_ to the five separate enzymatic complexes in the mitochondrial membrane. Oxygen is the final receptor of electrons at the end of the respiratory chain, where it is ultimately reduced to water. However, during this process, normally 2–3% of oxygen is not completely reduced and leads to the formation of reactive oxygen species (ROS), particularly, superoxide (${\text{O}}_{2}^{-}$, 16). Superoxide is mainly generated by coenzyme Q10 (CoQ10) partially reduced – and by complex III in the respiratory chain (Lenaz, 2001). Consequently, superoxide can be converted into hydrogen peroxide by superoxide dismutase in the mitochondria (Wang and Walsh, 1996).

Considering the demand for oxygen and energy required for normal placental development, it is logical to assume that any alterations at this level might compromise the overall process of placentation. Indeed, observational studies have demonstrated a higher incidence of preeclampsia in a family with mitochondrial dysfunction (Torbergsen et al., 1989). In addition, in women with preeclampsia (Folgerø et al., 1996) and their first-degree relatives, there is an abnormality in the expression of mitochondrial genes responsible for energy production, such as cytochrome C oxidase (Matsubara et al., 1997), or electron exchange processes preferentially favor oxidation (Wang and Walsh, 1998). On the contrary, it is well known that hypoxic conditions, for example, living at an altitude, lead to major susceptibility to the development of preeclampsia (Zamudio, 2003). In this sense, there is significant evidence showing that increased generation of ROS plays a key role in the development of preeclampsia (Davidge et al., 1992; Branch et al., 1994; Mikhail et al., 1994; López-Jaramillo et al., 1998; Little and Gladen, 1999; Staff et al., 1999; Sikkema et al., 2001). Reports have also confirmed that the origin of ROS is most likely from the placenta and, in particular, mitochondria (Wang and Walsh, 1998). A previous study reported that the number of mitochondria in placental tissue is far greater in women with preeclampsia and that these mitochondria have greater susceptibility to lipid peroxidation (Wang and Walsh, 1998); however, these observations were made in only a small number of patients (Table 1).

**Table 1:** Studies involving the activity of the mitochondria during preeclampsia.

Study	Relevant findings
Torbergsen et al., 1989	Features of preeclampsia as disturbed ion transport and prostaglandin synthesis, vasoconstriction, platelet aggregation, and hyperuricemia may be explain by mitochondrial dysfunction.
Folgerø et al., 1996	Mutations in mitochondrial transfer ribonucleic acid genes in two families are associated with a high occurrence of preeclampsia and eclampsia.
Matsubara et al., 1997	Number of mitochondria positive for COX staining markedly decreased in the placentae of the women with preeclampsia.
	Dysfunction of trophoblast’s mitochondria may be present in placenta of patients with preeclampsia.
Wang and Walsh, 1998	Increase of the amount of placental mitochondria in preeclampsia.
	Mitochondrial lipid peroxidation increased in preeclampsia.
	Mitochondrial generation of superoxide an important source of oxidative stress in preeclampsia.
Staff et al., 1999	The content of free 8-iso-prostaglandin F2a in decidual tissue from preeclampsia was significantly elevated than in control tissue.
Sikkema et al., 2001	Oxidative stress in placenta of preeclamptic women is increased.
Zamudio, 2003	Placenta from women living at high altitude has increased villous vascularization and thinning of the villous membranes, that together increases oxygen diffusion capacity.

In this sense, CoQ10, the only non-polar electron transporter into the mitochondrial respiratory chain, plays a key role in both production of energy and formation of ROS (Davidson, 1994; Lenaz, 2001). Circulating CoQ10 is known to act as a potent antioxidant, either directly (Kaikkonen et al., 1999) or via the regeneration of vitamin E (Hodson and Watts, 2003). Therefore, it seems logical to suggest that CoQ10 could be involved in the pathogenesis of preeclampsia.

## Coenzyme Q10 During Normal Pregnancy and Preeclampsia

The CoQ10 or ubiquinone, is a fat-soluble molecule synthesized endogenously from phenylalanine (benzoquinone ring) and mevalonic acid (responsible for isoprenoid units) and with a small contribution derived from the diet (Davidson, 1994). The CoQ10 participates in energy generation and plays a key role in mitochondrial respiration, as it is responsible for electron transport from complex I and II to complex III (Davidson, 1994). Thus, any alteration in the mitochondrial CoQ10 might result in reduced formation of energy and an increase in generation of ROS (Lenaz, 2001). On the contrary, CoQ10 acts as an antioxidant for lipoproteins, both membrane-bound and circulating (Hodson and Watts, 2003). In the later environment, the function of CoQ10 is related to its capability to increase the bioavailability of vasoactive substances such as nitric oxide (NO; Hodson and Watts, 2003; Teran, 2003).

Only a few studies have investigated CoQ10 during human pregnancy (Noia et al., 1996, 1998). These studies showed that the levels of CoQ10 increase progressively, starting in the first trimester and continue to rise until delivery (Table 2). In 2003, the authors’ group reported that Ecuadorian normal pregnant women, although with recognized nutritional deficiencies, had CoQ10 levels within the normal range as reported for other populations, but higher than non-pregnant women (Teran et al., 2003). However, the authors reported for the first time that women with preeclampsia show a significant reduction in CoQ10 levels (Teran et al., 2003), a result that was later confirmed by other authors (Palan et al., 2004). At that time, our working hypothesis was that during preeclampsia, there may be a “mechanism” that consumes CoQ10 (not related to diet), which might arise because of increased production of ROS. To investigate further this possibility, the author’s group investigated CoQ10 levels in the placenta and the umbilical cord and interestingly found that the levels were significantly higher in women with preeclampsia (Teran et al., 2005), suggesting a compensatory accumulation. However, all those studies were done in Quito, a high-altitude city (2800 m above the sea level). So, in a subsequent study, plasma CoQ10 was measured in Ecuadorian women living at sea level; results showed that normal pregnant women had significantly lower levels of CoQ10, but in those with preeclampsia, the difference was not as evident as in those patients living at high altitude; these observations may be related to the small sample size of this study (Teran et al., 2008). Interestingly, placental content of CoQ10 was also significantly higher in preeclamptic women at sea level (Teran et al., 2008). It was also consistent with a later study showing that CoQ10 levels in mitochondria from placentas of women with preeclampsia were significantly higher compared with mitochondria from normal placentas (Teran et al., 2007; Table 2).

**Table 2:** Studies involving Coenzyme Q10 (CoQ10) during normal pregnancy and preeclampsia.

Study	Relevant findings
Noia et al., 1996	Low CoQ10 levels in cases of spontaneous abortion. Increase in the plasma CoQ10 levels in relation to the contractile activity of the uterine muscle.
Noia et al., 1998	CoQ10 levels were higher in fetuses with hypoxia and non-immune hydrops.
Teran et al., 2003	Plasma CoQ10 levels were significantly higher in normal pregnant women in comparison to non-pregnant women. During preeclampsia there is a significant decrease in plasma levels of CoQ10 compared to normal pregnant women.
Palan et al., 2004	In pre-eclampsia there is decreased levels of CoQ10 and alpha-tocopherol, reducing the ability of antioxidant defense leading to the endothelial cell damage observed in preeclampsia.
Teran et al., 2005	CoQ10 in placenta and umbilical cord blood from women with preeclampsia was significantly higher compare to normal pregnancy.
Teran et al., 2008	Plasma and placental CoQ10 levels in normal pregnant women at sea level were significantly lower than in those living at high altitude. Preeclamptic women displayed higher placental CoQ10 content, which was only significant among those living at sea level; while CoQ10 plasma levels were significantly lower only in preeclamptic women living at high altitude.
Teran et al., 2009	Double blind, placebo controlled clinical trial with CoQ10 supplementation (200 mg/daily) from week 20 of pregnancy. Thirty women (25.6%) in the placebo group compared with 17 women (14.4%) in the CoQ10 group developed preeclampsia (*p* = 0.035).
Hernandez et al., 2017	At week 20 of pregnancy, plasma CoQ10 levels showed no difference between the control and supplemented groups. In the CoQ10 group women who developed preeclampsia showed significantly higher placental levels than normal pregnant women did. In mitochondria from preeclamptic women, levels of CoQ10 were no different among those in the placebo and CoQ10 groups.

According to our present data, it is likely that women with preeclampsia have already-established alterations in their levels of CoQ10 compared with normal pregnant women; these changes occur both in the plasma and the placenta. However, they occur to a greater extent in the mitochondria. However, all of these previous studies were conducted in women, who had already developed the disease; consequently, it was not possible to determine if a reduction in CoQ10 was a cause or a consequence. With this information, we decided to set up a randomized, double-blinded, and placebo-controlled clinical trial providing 200 mg of CoQ10 daily to 235 pregnant women starting from week 20 up to delivery; we then determined the rate of preeclampsia in this cohort of patients. At the end of the study, there were more patients in the preeclampsia placebo group (25.6% vs. 14.4%), demonstrating, also for the first time, that supplementation of CoQ10 represents an effective intervention to reduce the risk of developing preeclampsia (Teran et al., 2009).

*Post hoc* analysis showed that before supplementation, at week 20 of pregnancy, plasma CoQ10 levels showed no difference between controls and the supplemented groups. Interestingly, at delivery, placental tissue showed no differences in the placebo group when compared between women with normal pregnancy and those with preeclampsia, while in the CoQ10 group, women with preeclampsia showed significantly higher placental levels compared with normal pregnant women. However, mitochondrial levels of CoQ10 in the placenta from pregnant women with preeclampsia receiving placebo did not show significant differences when compared with those receiving supplementation of CoQ10. These results suggest that in women with preeclampsia, although CoQ10 reduced preeclampsia and was present in high levels in placental tissue, the mitochondrial levels of CoQ10 did not change significantly (Hernandez et al., 2017).

## Conclusion

**FIGURE 1:**
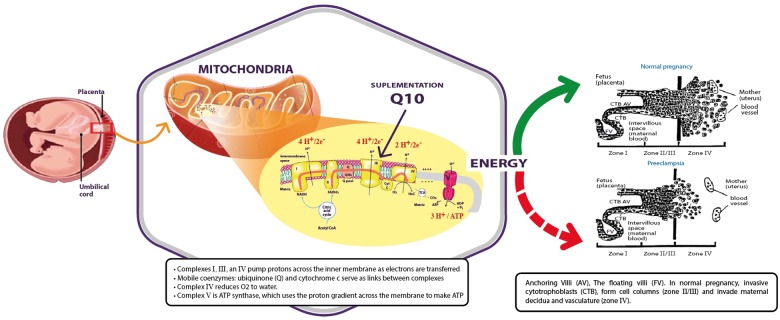
Mitochondria produce energy for placental development during normal pregnancy (green arrow) that is abnormal in preeclamptic women (red dashed arrow). Supplementation of coenzyme Q10 might restore the activity of the mitochondria.

In conclusion, preeclampsia is associated with abnormal placental development as a result of several factors; of these, the dysfunction of the mitochondria appears to be the most important. In that case, the lack of energy and the associated increase of free radicals might be due to the deficiency or the consumption of CoQ10, as supplementation of CoQ10 was shown to be an effective intervention for the reduction of the rate of preeclampsia (Figure 1).

## Author Contributions

ET, IH, and AC contributed to the conception and design of the studies. LT and ST organized the databases. ET, ST, and AC performed the statistical analysis. ET, IH, and ST wrote the first draft of the manuscript. C-GH, MS-M, and GM wrote sections of the manuscript. All authors contributed to manuscript revision, read, and approved of the submitted version.

## Conflict of Interest Statement

The authors declare that the research was conducted in the absence of any commercial or financial relationships that could be construed as a potential conflict of interest.
